# Cannabinoid Type 1 Receptors Transiently Silence Glutamatergic Nerve Terminals of Cultured Cerebellar Granule Cells

**DOI:** 10.1371/journal.pone.0088594

**Published:** 2014-02-12

**Authors:** Jorge Ramírez-Franco, David Bartolomé-Martín, Beatris Alonso, Magdalena Torres, José Sánchez-Prieto

**Affiliations:** Departamento de Bioquímica, Facultad de Veterinaria, Universidad Complutense, Madrid, Spain; UCL School of Pharmacy, United Kingdom

## Abstract

Cannabinoid receptors are the most abundant G protein-coupled receptors in the brain and they mediate retrograde short-term inhibition of neurotransmitter release, as well as long-term depression of synaptic transmission at many excitatory synapses. The induction of presynaptically silent synapses is a means of modulating synaptic strength, which is important for synaptic plasticity. Persistent activation of cannabinoid type 1 receptors (CB1Rs) mutes GABAergic terminals, although it is unclear if CB1Rs can also induce silencing at glutamatergic synapses. Cerebellar granule cells were transfected with VGLUT1-pHluorin to visualise the exo-endocytotic cycle. We found that prolonged stimulation (10 min) of cannabinoid receptors with the agonist HU-210 induces the silencing of previously active synapses. However, the presynaptic silencing induced by HU-210 is transient as it reverses after 20 min. cAMP with forskolin prevented CB1R-induced synaptic silencing, via activation of the Exchange Protein directly Activated by cAMP (Epac). Furthermore, Epac activation accelerated awakening of already silent boutons. Electron microscopy revealed that silencing was associated with synaptic vesicle (SV) redistribution within the nerve terminal, which diminished the number of vesicles close to the active zone of the plasma membrane. Finally, by combining functional and immunocytochemical approaches, we observed a strong correlation between the release capacity of the nerve terminals and RIM1α protein content, but not that of Munc13-1 protein. These results suggest that prolonged stimulation of cannabinoid receptors can transiently silence glutamatergic nerve terminals.

## Introduction

Endocannabinoids are activity-dependent retrograde messengers that are widely involved in regulating synaptic transmission throughout the mammalian central nervous system (CNS). Postsynaptically-released endocannabinoids travel backwards across the synapse to activate presynaptic type 1 cannabinoid receptors (CB1Rs: [1]). CB1Rs are G protein-coupled receptors that transiently suppress neurotransmitter release at glutamatergic synapses [2]. Short-term effects of CB1R are attributed to the inhibition of Ca^2+^ entry at nerve terminals [3] although the opening of K^+^ channels [4], [5] may also be involved. Cannabinoid receptors also mediate long lasting changes in synaptic strength and persistent activation of cannabinoid receptors induces long-term depression of synaptic transmission (LTD). Some forms of cannabinoid induced LTD are expressed presynaptically and involve alterations in the release machinery that produce a reduction in neurotransmitter release [6]–[8]. At other synapse, for example the parallel fiber to Purkinje cell synapses, cannabinoid-induced LTD is induced presynaptically via decreased neurotransmitter release, but expressed postsynaptically [9]

Synaptic silencing is a way to control synaptic strength with important implications in synaptic plasticity. Postsynaptically silent synapses [10], [11] are defined as those synapses in which presynaptic activation fails to induce a postsynaptic current under resting membrane potential, while postsynaptic activation occurs in depolarizing conditions (reviewed in [12]). This fact has been associated with the absence of AMPA receptors, whose activation depolarizes the membrane to release the magnesium block from NMDA receptors. Postsynaptically silent synapses have crucial roles in processes such as LTP (by recruiting previously inactive synapses) [13], [14] or development [15], [16]. It has been proposed that postsynaptic un-silencing is undistinguishable from classical LTP [12]. However, in the last decade, presynaptic silencing has emerged as a mechanism for synaptic strength modulation. Presynaptically silent synapses are synapses containing full complement of exocytotic release proteins that fail to release neurotransmitter in response to a strong depolarization and Ca^2+^ influx [17], [18]. Silent synapses have a normal function of postsynaptic receptors [19], [20], but contain a diminished readily releasable pool of vesicles [19] and exhibit deficient vesicle priming, the process that renders vesicles fusion-competent [21]. In hippocampal excitatory neurons, the induction of silent synapses is observed after sustained depolarization [19] and after prolonged stimulation of G protein-coupled receptors (GPCRs), such as adenosine A_1_ and GABA_B_ receptors [22], although no evidence of CB1R-induced silencing was obtained in that study. In contrast, activation of CB1R by endogenous cannabinoids has been shown to silence GABAergic terminals in hippocampal slices [23]. Then, it is not known whether CB1R can induce presynaptic silencing at glutamatergic synapses and moreover, the signaling mechanisms underlying GPCR-mediated silencing remain poorly understood.

The fluorescent membrane marker FM1-43 that binds to synaptic vesicles after a fusion event has been used to detect presynaptically silent boutons [24]. Thus, some studies have considered presynaptic silent synaptic boutons those that fail to take FM1-43 but exhibiting a mature profile of synaptic proteins [17], [18]. However, FM1-43 is not a good tracer of all modes of neurotransmitter release. Thus, while full loss of fluorescent membrane dye is consistent with release events in which the synaptic vesicle undergoes full collapse, vesicular retention of the fluorescent membrane marker is observed when release occurs via “kiss-and-run” [25], [26], raising the possibility of a failure in dye uptake in those synapses undergoing kiss and run processes during the loading phase. Alternatively, presynaptic silencing can be measured with VGLUT1-pHluorin [27] expressed in dissociated cultured neurons. pHluorins have been useful to monitor vesicle turnover because its fluorescence is dim upon acidification of the synaptic vesicle, but increases during vesicular fusion [28]. Because the increase in fluorescence upon exocytosis is due to the release of protons after vesicle fusion, this VGLUT1-pHluorin allows to measure different forms of synaptic vesicle exocytosis including “kiss-and-run” [29]. An additional advantage of VGLUT1-pHluorin to study the mechanisms of presynaptic silencing is that the fluorescence change associated to a round exocytosis/endocytosis is highly reproducible after several stimulations [30]. Then, VGLUT1-pHluorin unequivocally detects silenced synaptic boutons as those active boutons that become unable to undergo exocytosis after applying the protocol that induces presynaptic silencing.

We have used VGLUT1-pHluorin expressed in dissociated cerebellar granule neurons to determine whether CB1 receptors induce presynaptic silencing. Here we show that prolonged stimulation (10 min) of CB1 receptors with the cannabinoid agonist HU-210 induces the silencing of previously active synapses in cultured cerebellar granule cells. However, the presynaptic silencing induced by HU-210 is a transient phenomenon as it reverses after 20 min. Synaptic silencing was prevented by increasing cAMP levels with forskolin and by activation of Exchange Protein directly Activated by cAMP (Epac), but not by activating the cAMP-dependent protein kinase (PKA). Electron microscopy revealed that synaptic silencing involves the redistribution of synaptic vesicles (SVs) within the nerve terminal, diminishing the number of vesicles close to the active zone (AZ). Finally, we observed a strong correlation between the release capacity of the nerve terminals and the RIM1α protein content.

## Materials and Methods

### Ethics Statement

All procedures relating to the care and use of animals were performed in accordance with the European Council Directives (86/609/EEC and 2010/63/EU) and with the guidelines established by the National Council on Animal Care and were approved on 21-1-2010 by “Comité de Experimentación Animal” the local Animal Care Committee of the Universidad Complutense de Madrid (UCM, Madrid, Spain). Every possible effort was made to minimize animal suffering and the number of animals used.

### Primary culture of cerebellar neurons

Primary dissociated cerebellar cultures were derived from the cerebellum of 7-day-old (P7) male or female Wistar albino rat pups, as described previously [31]. We euthanized pups using cervical dislocation. The cells were seeded onto poly-L-lysine-coated coverslips (1–3×10^5^ cell/coverslip), or in 96- or 6-well tissue culture plates (10^5^ or 3.5×10^6^ cells/well, respectively). Experiments were carried out between 7 and 10 DIV. This is a highly pure granule cell preparation, although it may contain some types of interneurons that always represent a small proportion (less than 10%) of cultured cerebellar neurons [32].

### Immunocytochemistry

Cerebellar granule cells at 7–10 DIV were fixed for 15 minutes at room temperature (RT) with 4% paraformaldehyde, washed and then permeabilized with PBS-0.2% Triton X-100 for 6 minutes. Before immunostaining, the cells were blocked for 1 hour at 37°C. The cells were then incubated overnight (o/n) at 4°C with the following primary antibodies: guinea pig polyclonal anti-CB1R (1∶300, Frontier Institute Co., Ltd.) and rabbit-polyclonal anti-synaptophysin-1 (1∶200, Synaptic Systems). Subsequently, the cells were washed and then incubated with Alexa (Molecular Probes, Invitrogen) labeled secondary antibodies (1∶200). After washing, coverslips were mounted with Prolong Antifade with DAPI (Invitrogen).

### FM1-43 Live Cell Imaging

The cells were incubated for 10 minutes in calcium-free, 5 mM potassium, HEPES buffer medium, (HBM), and then for 90 seconds with FM1-43 dye (10 µM; Invitrogen) in high potassium (50 mM KCl, 1.33 mM CaCl_2_) buffer. The cells were then perfused for 10 minutes with calcium-free, 5 mM potassium HBM buffer to remove the surface-bound dye, and during this period the cells were exposed to the cannabinoid agonist HU-210 (5 µM; Tocris Bioscience). FM1-43 unloading was induced by depolarization with high potassium (50 mM KCl) first during 10 seconds and then during 2 minutes. (Images were obtained at an acquisition rate of 1 Hz on a Nikon Eclipse TE2000-S microscope equipped with a Nikon CFI Plan Apo VC 60X Oil 1.4 (NA) and a CCD camera (iXon^EM^+DU885, Andor Technology). A 479-nm monochromator was used for excitation and the emitted light was collected using a fluorescein isothiocyanate (FITC) filter.

### Analysis of FM1-43 experiments

FM1-43 experiments were analyzed as described previously [31]. Briefly, different fields were selected randomly and individual synaptic boutons were analyzed. Regions of interest (ROIs) were identified with Igor Pro software as previously described [33].

### VGLUT1-pHluorin Live Cell Imaging

The cell suspension was electroporated with Amaxa™ Nucleofector II using Rat Neuron Amaxa Nucleofector Kit (Lonza), following the instructions provided by the manufacturer (programme O-03) as described [34]. For transfection, 1 µg of VGLUT1-pHluorin (VGpH) DNA [27] was used per 5×10^6^ cells and the cells were finally seeded at a density of 1.6×10^6^ cells/coverslip. Experiments were performed at 7 DIV. Initially, the cells were incubated for 10 minutes in calcium-free and 5 mM-potassium HBM buffer. For VGpH double pulse experiments, a baseline in low potassium buffer (30 sec) was followed by a stimulation (50 mM KCl, 10 sec) and by a recovery period with 5 mM-potassium buffer (1 min). The cells were maintained at rest for 10 minutes and where indicated, they were incubated with 5 µM HU-210 during this period. Next, the experimental protocol described in step 1 (30 second baseline, 10 second stimulation and 1 minute of recovery) was repeated, followed by a short pulse (30 seconds) of NH_4_Cl, in order to estimate the maximal signal. For triple pulse experiments the protocol used involved a 30 second baseline recording, followed by a 10 second depolarization pulse and a 1 minute recovery period, after which NH_4_Cl (50 mM) was perfused in each pulse. The cells were maintained at rest for 10 minutes between pulses and, where indicated, they were treated with HU-210 for 40 seconds during the baseline period or for 10 minutes between pulses. For single pulse experiments the cells were maintained for 10 minutes at rest and they were then incubated with different drugs, as indicated in the figure legend. Images were captured at an acquisition rate of 4 Hz and averaged into one single frame per second to enhance the signal-to-noise ratio, resulting in an actual readout of 1 Hz.

### Analysis of VGLUT1-pHluorin experiments

Images were analyzed using ImageJ software (http://rsbweb.nih.gov/ij). For image processing of VGpH experiments, background subtraction was carried out by averaging several cell-free regions and subtracting this value from each individual ROI. ROIs were defined manually as NH_4_Cl-responsive puncta with a diameter of over 1 µm. Somatic regions were excluded from the analysis. After background subtraction, the averaged value of the ten first frames of each ROI was subtracted to calculate the zero value during baseline. The maximal signal during NH_4_Cl perfusion was used to normalize each trace, resulting in values ranging from zero to 1. Boutons were classified as silent boutons when the signal elicited by 50 mM KCl perfusion was less than 4% of the ammonium chloride signal. This threshold was selected as it corresponds to the average noise level of individual traces.

### Electron microscopy

To visualize the SV arrangement within the presynaptic terminals, cultured cerebellar granule cells were obtained from wild type rats and cortical synaptosomes were obtained from wild type or *cnr1^−/−^* mice [35] as described previously [36], and they were incubated for 10 minutes with low-potassium HBM buffer in the presence or absence of 5 µM HU-210. Next, cells were washed and then fixed for 2 hours at 4°C with 4% paraformaldehyde/2.5% glutaraldehyde in Millonig's sodium phosphate buffer (0.1 M, pH 7.3). The cells were then washed twice and incubated overnight at 4°C in Millonig's buffer, after which they were post-fixed in 1% OsO_4_/1.5% K_3_Fe(CN)_6_ for 1 hour at RT and dehydrated in ethanol. To detach the cell layer from the plate, cells were treated with propylene oxide and the entire monolayer was collected and embedded using the SPURR embedding kit (TAAB, Aldermaston, England). Ultrathin sections (70 nm) were routinely stained with uranyl acetate and lead citrate, and images were obtained using a JEM 1010 transmission electron microscope. Measurements were made with ImageJ software. The relative percentage of SVs per AZ were calculated in 10 nm bins from the membrane at the AZ, using the outer membrane of the SVs and the inner layer of the AZ plasma membrane as reference points. The mean number of SVs in the first 10 nm from the AZ and the total number of vesicles per synaptic terminal were also determined. Only synaptosomes with a notable postsynaptic membrane (synaptoneurosomes) were analyzed [37].

### Post hoc immunocytochemistry

To identify the field analyzed in functional (FM1-43) experiments, the chambers subjected to post-hoc immunocytochemistry were marked to ensure that they were re-positioned in the same location. After FM1-43 unloading, the cells were processed as described above. The following antibodies were used at the indicated concentrations: guinea pig polyclonal anti-CB1R (1∶300; Frontier Institute Co., Ltd.) and mouse monoclonal anti-Munc 13-1, (1∶1000; Synaptic Systems) or rabbit polyclonal anti-RIM1 (1∶400; Synaptic Systems). The coverslips were then washed and incubated with Alexa labeled secondary antibodies (1∶200; all from Molecular Probes, Invitrogen). After several washes in PBS, the coverslips were mounted with Prolong Antifade with DAPI (Invitrogen). The exact field analyzed in the experiment was located using a serial reconstruction of phase contrast images as a reference.

For quantitative analysis, all images were acquired at identical light intensity, electronic gain and exposure time for each marker. Background subtraction was performed using the ImageJ plugin, based on the rolling ball radius algorithm [38] and applying a radius of 12 pixels for all the images. Spurious ROIs were manually removed. The mask generated in the FM1-43 experiment using Igor Pro software was superimposed onto the immunocytochemistry images using the “Align RGB planes” tool. The ROIs located at the edges of the image, did not exactly match the immunoreactive puncta and they were therefore excluded from the quantification. To appropriately quantify staining and to correlate the intensity of individual synaptic terminal staining with the amount of protein (although these two parameters are not linearly related), staining intensities were measured as grey-scale values (16 bits) using ImageJ software. The integrated density value was calculated as the sum of the grey values of each of the individual pixels defined in a given ROI. The functional responses of FM1-43 were blindly sorted into groups according to their immunoreactivity (IR) values after normalizing all the IR value of a given field to the corresponding mean IR value. When sorting according to the RIM1α/CB1R ratio or the Munc13-1/CB1R ratio, the quotient between the normalized IRs was used.

### cAMP accumulation

Cerebellar granule cells (10 DIV; 10^5^ cells/well in 96-well tissue culture plates) were incubated in 5 mM potassium HBM buffer plus 1 mM IBMX (Sigma-Aldrich), and 5 µM HU-210 or 1 µM forskolin were added where indicated. After incubation for 30 minutes at 37°C, the cells were lysed and the cAMP content was measured with the cAMP dynamic 2 detection kit (CisBio), using a FLUOstar Omega microplate reader (BMG LabTech) equipped with the Advanced Assay Technology package for time-resolved Förster resonance energy transfer (TR-FRET) applications.

### Statistical analysis

The data were analyzed using Statgraphic, OriginPro 8.0 or SigmaPlot 10 software. The specific test applied in each case is indicated in the figure legend or in the text. The data are represented as the mean ± S.E.M.: *p<0.05, **p<0.01. Differences were considered statistically significant when p<0.05 with a confidence limit of 95%.

## Results

**Figure 1:**
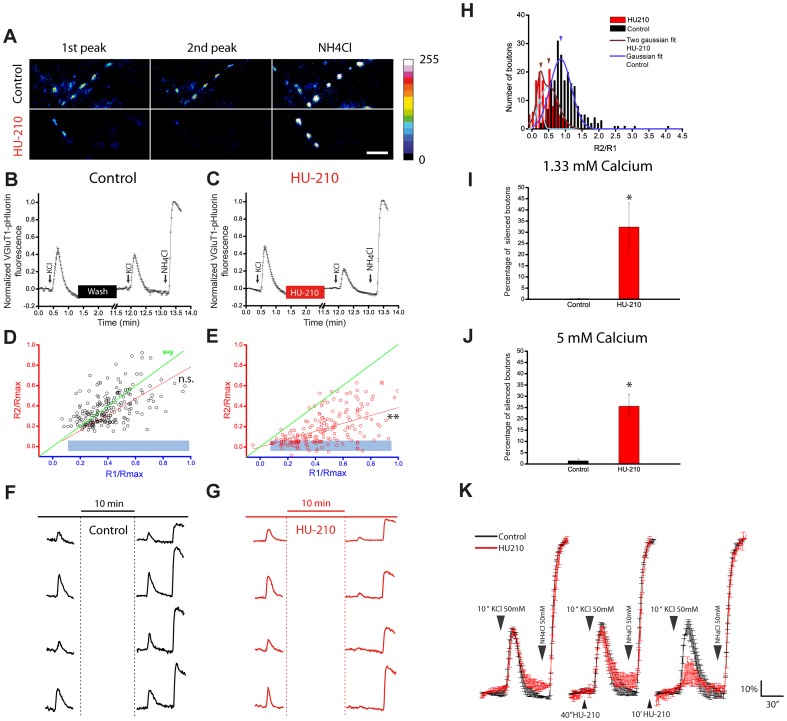
VGLUT1-pHluorin reveals presynaptically silent synapses after prolonged stimulation of cannabinoid receptors . A) Representative fluorescence images of synaptic boutons after two stimulations with 50 mM KCl (10 sec) followed by NH_4_Cl (50 mM) perfusion in control and after HU-210 (5 µM, 10 min) added between the first and the second peak. B, C) Mean response of all the individual nerve terminals analyzed in double pulse experiments, in control and HU-210, respectively. D, E) Relationship between the first and the second response of the individual synaptic boutons averaged in B and C, in control and HU-210 treated neurons, respectively. The light blue rectangle denotes the silenced synaptic boutons. F, G) Examples of some individual traces of the experiments shown in B and C. H) Histogram showing the bi-modal distribution of the responses' ratios after HU-210 treatment; fitting in brown (HU-210) and dark blue (control); light blue is the individual fitting for each of the populations in HU-210 treatment. Percentage of silenced synaptic boutons at 1.33 mM extracellular Ca^2+^ (I) (Control: 0.25±0.25% vs HU-210: 32.31±10.86%; p<0.05) and at 5.0 mM extracellular Ca^2+^ (J) (Control: 1.4±0.8% vs HU-210: 25.61±5.3%; p<0.05). K) Average response of triple pulse experiment showing the requirement of a long HU-210 incubation period (10 min) in order to induce silencing, notice the absence of inhibition after a 40″ exposition to the receptor agonist. Number of synaptic boutons analyzed (total synaptic boutons/ silent and active, n, number of coverslip). In control at 1.3 mM Ca^2+^: 209/1 and 208, n = 4. In HU-210 at 1.3 mM Ca^2+^: 195/63 and 132, n = 4. In control at 5.0 mM Ca^2+^: 157/2 and 155, n = 4. In HU-210 at 5.0 mM Ca^2+^: 134/35 and 99, n = 4.

Persistently active cannabinoid receptors mute a subpopulation of hippocampal interneurons [23]. To determine whether the cannabinoid agonist HU-210 converts functional synaptic boutons into silent ones at excitatory synapses we transfected cerebellar granule cells with VGLUT1-pHluorin to visualise several rounds of the exo-endocytotic cycle. VGLUT1-pHluorin fluorescence is quenched at the acidic intravesicular pH, while is maximal at alkaline pH. After depolarization (50 mM KCl, 10 sec), exocytosis is visualised as an increase in fluorescence due to exposure of VGLUT1-pHluorin to extracellular neutral medium followed by a decrease phase due to compensatory endocytosis and vesicular lumen acidification. Alkalinization of the entire synaptic vesicle population with NH_4_Cl generates maximum fluorescence. This stimulation protocol recycles a synaptic pool fraction around 40%, similar to that found in hippocampal neurons [39]. In double pulse experiments, two stimulations with 50 mM KCl were applied, separated by a 10 min washout period and the responses of all individual nerve terminals analysed were averaged. In control conditions at 1.3 mM Ca^2+^, two KCl depolarization pulses resulted in comparable increases in fluorescence, with average peak values of 44.0±4.0% and 39.0±1.0% of the NH_4_Cl signal, respectively (Fig. 1A,B). However, treatment with HU-210 after the first KCl pulse strongly reduced the response to the second pulse (47.1±2.0% and 21.8±1.0%, respectively: Fig. 1A,C). Accordingly, the distribution of the peak fluorescence of individual nerve terminals was similar for the two peaks in control conditions (Fig. 1D,F), while a strong reduction in the fluorescence of the second peak was observed in HU-210 treated cells (Fig. 1E,G). This distribution indicates that many nerve terminals that responded to the first stimulation did not exhibit a similar increase in fluorescence when stimulated after HU-210 administration (Fig. 1E,G) giving rise to a bimodal distribution of the response ratio (Fig 1H). Nerve terminals whose response to a second KCl pulse was less that 10% of the average control responses (approx 4% of the NH_4_Cl response) were identified as silent synapses. This threshold was selected as it corresponds to the average noise level of individual traces. Indeed, the proportion of silent synaptic boutons in control conditions (0.25±0.25%) increased to 32.3±10.9% after HU-210 treatment (Fig. 1I). Increasing the Ca^2+^ concentration of the extracellular medium to 5 mM did not prevent this silencing by HU-210 treatment (Control, 1.4±0.8%; HU-210, 25.6±5.3%: Fig. 1J). Triple pulse VGLUT1-pHluorin experiments demonstrated that the induction of presynaptic silencing requires the persistent activation of cannabinoid receptors. Treatment of cells for 40 sec with HU-210 had no effect on the average response to KCl, indicating no evidence of synaptic silencing (Fig. 1K). However, when incubated with the agonist for 10 min, the fluorescence response of these same synaptic boutons was weaker (Fig. 1J). In control cells, the three KCl pulses resulted in responses of a similar magnitude.

**Figure 2:**
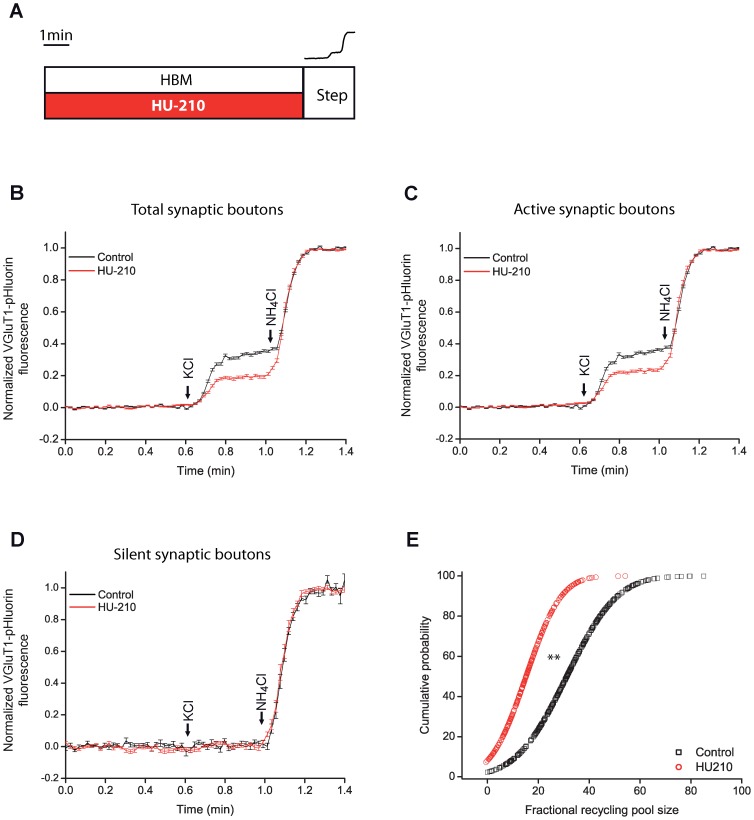
HU-210 reduced the size of the SV recycling pool in VGLUT1-pHluorin experiments with bafilomycin. Control cells in HBM and HU-210 (5 µM, 10 min) treated cells, were stimulated with 50 mM KCl (10 sec) followed by the addition of NH_4_Cl (50 mM) (step) as shown in scheme A. Average response of the whole population (B) and of the active (C) and silent (D) subpopulations of synaptic boutons, at 1.33 mM extracellular Ca^2+^. E) Cumulative probability plot of individual responses in control (black squares) and HU-210 treated cells (red circles). Number of synaptic boutons analyzed (total/ silent and active synaptic boutons, n, coverslip number) in each condition. In control: 402/10 and 392, n = 4. In HU-210: 267/45 and 222, n = 4. **p<0.01, Kolmogorov-Smirnov test.

Bafilomycin is a specific inhibitor of vacuolar-type H^+^ATPase that prevents the acidification of synaptic vesicles and therefore, the decay phase of the KCl-induced changes in fluorescence. Bafilomycin then allows the estimation of net exocytosis, and hence the size of the recycling pool. At 1.3 mM Ca^2+^, HU-210 reduced the size of the SV recycling pool as shown in the average response of the whole population of synaptic boutons (Fig 2B). This response results from a reduction in the recycling pool of active synaptic boutons (Fig 2C) and from an increase in the percentage of silent synaptic boutons as shown in the cumulative probability distribution (Fig 2D,E) (1.9±0.6% in control and 19.1±5.2% in HU-210-treated cells, p<0.05 compared to control).

**Figure 3:**
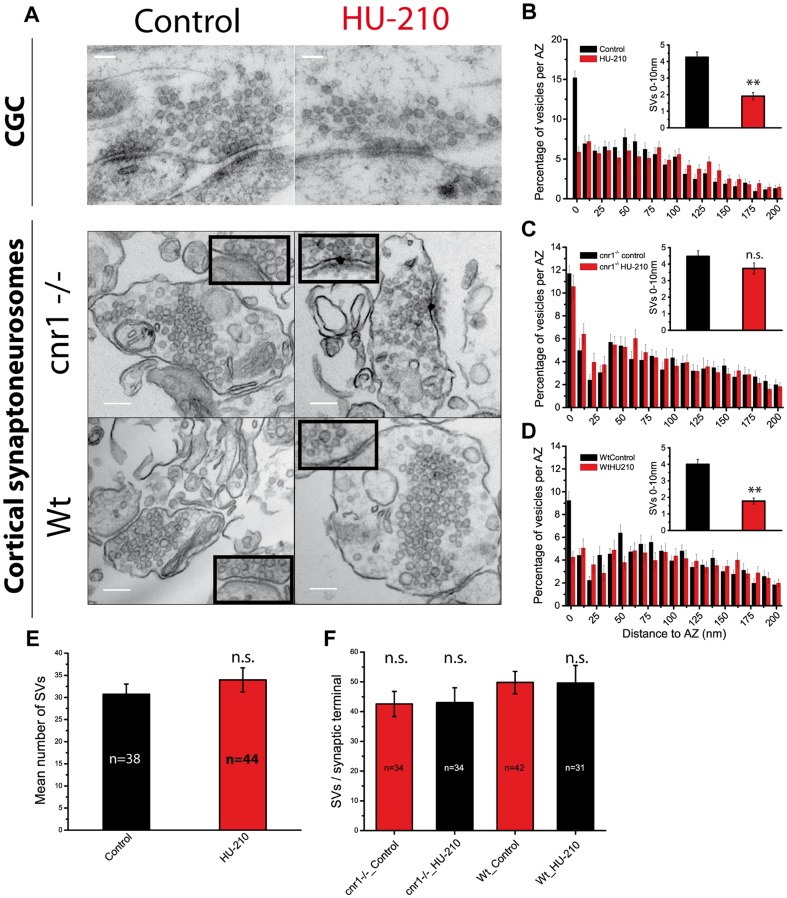
HU-210 treatment induced synaptic vesicles retraction from the active zone plasma membrane. A) Electron micrographs of cerebellar granule cells (top panel) and cortical *cnr1* ^-/-^ (middle panel) and wild type (bottom panel) synaptoneurosomes in control conditions (left) and after HU-210 (5 µM, 10 min) (right). B,C,D) Quantification of the spatial distribution of the synaptic vesicles per active zone in Cerebellar Granule Cells, *cnr1* ^-/-^ and wt synaptoneurosomes, respectively. The % of SVs closer than 10 nm to the AZ membrane were: in CGC, control 15.20±0.85% vs HU-210 5.87±0.66%; p<0.01, Student's t-test; in *cnr1* ^−/−^ synaptoneurosomes, control 11.70±0.67% vs HU-210 10.56±1.01%; p>0.05, Student's t-test; in wt synaptoneurosomes, control 9.24±0.75% vs HU-210 4.27±0.46%; p<0.01, Student's t-test. The insets represent the mean number of SVs closer than 10 nm to the AZ membrane in each condition: In Cerebellar Granule Cells, 4.26±0.31 (control) vs 1.90±0.22 (HU-210); p<0.01, Student's t-test; in *cnr1* ^−/−^ synaptoneurosomes, 4.46±0.33 (control) vs 3.73±0.34 (HU-210); p>0.05, Student's t-test; in wt synaptoneurosomes, 4.0±0.29 (control) vs 1.77±0.18 (HU-210); p<0.01, Student's t-test). Mean number of SVs per synapse in cerebellar granule cells (E) and in cortical synaptoneurosomes (F). Scale bar in CGC 100 nm and in synaptoneurosomes 150 nm.

Exocytosis of synaptic vesicles close to the presynaptic membrane is triggered by increases in Ca^2+^ in the active zone. Since presynaptic silencing persists at high (5 mM) extracellular Ca^2+^ concentrations it argues against a reduction in Ca^2+^ influx as the main cause of the deficit in exocytosis, suggesting a possible change in the distribution of SVs. Electron microscopy studies revealed that exposing cells to HU-210 did not alter the number of SVs per synaptic bouton (30.7±2.3 in control and 33.9±2.7 in HU-210-treated cells: Fig. 3E,F), although HU-210 did significantly reduce the number of vesicles in close proximity (<10 nm) to the presynaptic membrane (4.3±0.3 SVs in control and 1.9±0.2 in HU-210-treated cells: Fig. 3A,B, inset). This fact resembled some of the ultra-structural defects observed in docking mutants [40].

To demonstrate that this change in the distribution of SVs induced by HU-210 also occurs in brain tissue, we repeated the experiment using cerebrocortical synaptosomes and we specifically analyzed synaptoneurosomes (nerve terminals that contain an attached postsynaptic structure). Despite other limitations, cortical synaptoneurosomes have two major advantages for ultrastructural studies: i) they represent native brain tissue and ii) they preserve most of their ultrastructural features when a post-synaptic membrane is apposed (synaptoneurosomes) in particular the distribution of SVs [37]. HU-210 reduced the number of synaptic vesicles that are close to presynaptic membrane of synaptoneurosomes (4.0±0.3 SVs in control and 1.8±0.2 in HU-210-treated synaptoneurosomes: Fig. 3A,D, inset). These changes in vesicle distribution were not observed in synaptoneurosomes obtained from mice lacking CB1 receptors (cnr1^−/−^: 4.5±0.3 in control and 3.7±0.3 in HU-210-treated synaptoneurosomes: Fig. 3A,C, inset).

**Figure 4:**
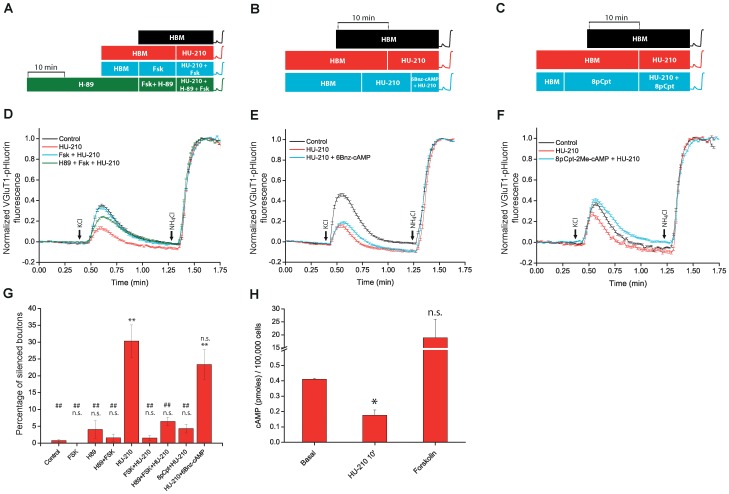
Epac protein activation largely prevents HU-210-induced presynaptic silencing. A, B, and C, schemes showing the sequence of drug additions in control; HU-210 (5 µM, 10 min); Forskolin (50 µM, 15 min) either alone and with HU-210; H-89 (10 µM, 30 min) either alone, with forskolin and with forskolin and HU-210; 6Bnz-cAMP (200 µM, 5 min) and HU-210; and 8p-CPT (50 µM,15 min) either alone and with HU-210. After these treatments cells were stimulated with 50 mM KCl (10 sec) followed by the addition of NH_4_Cl (50 mM). D,E and F, show average responses of total synaptic bouton populations under different conditions. G) Percentage of silent synapses in each condition. H) cAMP levels in cerebellar granule cells in basal conditions, after HU-210 (5 µM, 10 min); and after forskolin (50 µM, 15 min). Multiple comparison ANOVA followed by Bonferroni's test for means comparison was applied to data in panel G, **p<0.01 vs control; ##p<0.01 vs HU-210. For statistical significance of data shown in Fig H, Welch's t-test was used. ** p<0.05 vs basal cAMP levels. Number of synaptic boutons analyzed (total/ silent and active synaptic boutons, n, coverslip number). In panel D, Control: 169/2 and 167, n = 3. HU-210: 159/56 and 103, n = 5. Fsk+HU-210: 269/4 and 265, n = 4. H89+Fsk+HU-210: 417/29 and 388, n = 7. In panel E, Control: 138/0 and 138, n = 2. HU-210: 117/21 and 96, n = 2. HU-210+6Bnz-cAMP: 305/54 and 251, n = 7. In panel F, Control: 183/5 and 178, n = 4. HU-210: 198/70 and 128,n = 6. 8pCpt+HU-210: 144/6 and 138, n = 5.

The persistent activation of cannabinoid receptors appears to initiate an intracellular signalling cascade that results in the retraction of SVs located close to the presynaptic membrane. As a consequence, the depolarization-induced Ca^2+^ influx fails to induce exocytosis. However, the signalling mechanisms underlying CB1R-induced presynaptic silencing remain unknown. Cannabinoid receptor activation inhibits adenylyl cyclase and reduces cAMP levels in the cell [5], [41], while cAMP accelerates SV recycling [42]. One possibility is that presynaptic silencing is mediated by a decrease in cAMP levels. We observed that prior incubation of cerebellar granule cells with the adenylate cyclase activator forskolin fully prevented HU-210-induced presynaptic silencing as shown in (Fig. 4A,D,G) where the average response of all individual nerve terminals were analysed. The effect of forskolin was also observed in the presence of the protein kinase A inhibitor H-89 (10 µM, 50 min), arguing against a major role for this kinase in the prevention of silencing. Furthermore, 6-Bnz-cAMP, a specific activator of PKA, showed limited activity in reversing the silencing effect of the cannabinoid receptor agonist, suggesting a minor role of PKA in preventing the HU-210-induced presynaptic silencing (Fig. 4B,E,G). In addition to PKA, several other signalling pathways can be activated by cAMP. Epac1 and Epac2 are cAMP-dependent guanine nucleotide exchange factors for the small GTPases Rap1 and Rap2, which are important mediators of the effects of cAMP [43]. Significantly, we found that the specific membrane-permeable Epac activator 8-pCPT-2′-O-Me-cAMP (8pCpt) also prevented HU-210-induced silencing (Fig. 4C,F,G). Moreover, in parallel experiments HU-210 reduced the overall basal cAMP cellular levels from 0.41±0.01 pmol to 0.18±0.03 (Fig. 4H).

**Figure 5:**
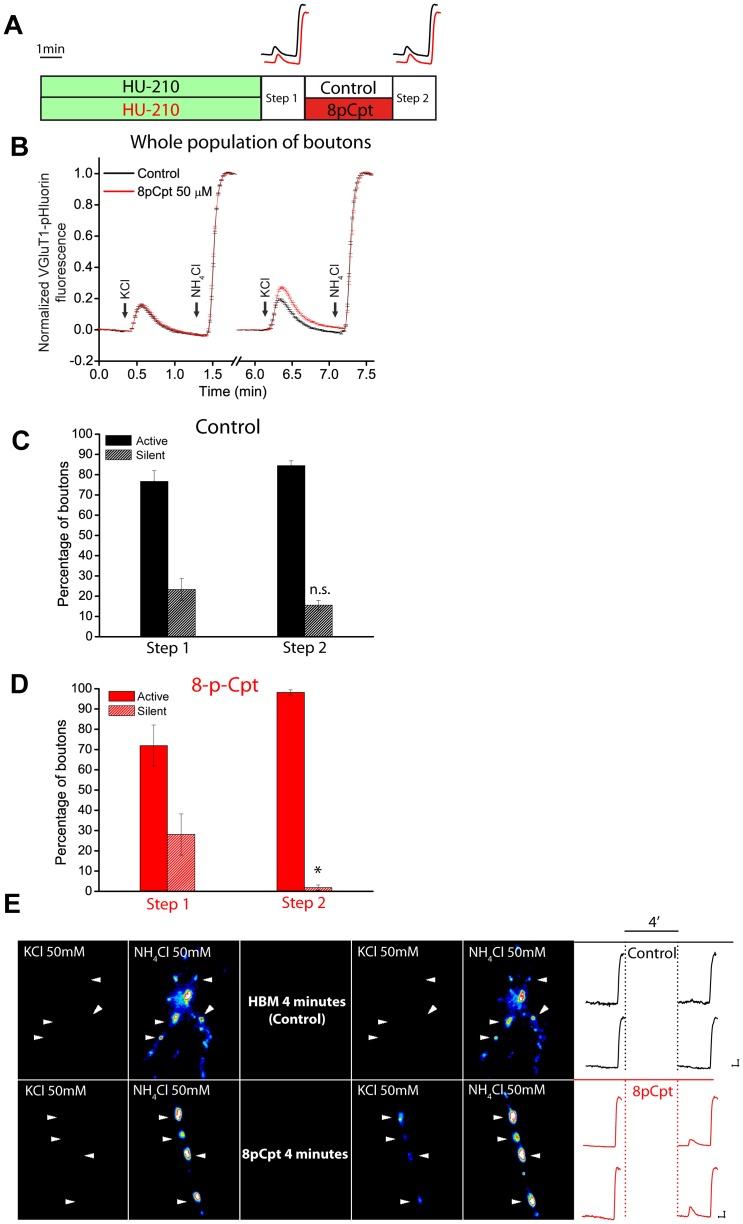
Epac protein activation accelerates awakening of HU-210-induced silent synaptic boutons. A) scheme showing the cell treatment. Cerebellar granule cells were exposed to HU-210 (5 µM, 10 min), and stimulated with KCl (50 mM, 10 sec) followed by perfusion of NH_4_Cl (50 mM) (step 1) and silent and active synaptic boutons were determined. Then, cells were treated with the Epac activator 8p-Cpt (50 µM, 4 min; red traces), or with HBM (control; black traces) during the inter-step period, and silent and active synaptic boutons were again quantified (step 2). B) Average responses of the whole population of synaptic boutons are shown for each treatment. Diagrams showing the percentages of silent and awakened synaptic boutons observed after the second pulse in control (C) and in 8-pCpt treated cells (D). E) representative fluorescence images of synaptic boutons responding to two stimulations with 50 mM KCl (10 sec) followed by NH_4_Cl (50 mM) perfusion in control (HBM) and after 8p-CPT (50 µM, 4 min). Arrow heads indicate individual synaptic boutons. Two examples of individual responses in control (upper traces, black) and another two responses in 8p-CPT (lower traces, red) are also shown. Number of synaptic boutons analyzed (total / silent and active synaptic boutons, n, coverslip number). In control: 358/66 and 292, n = 7. In 8pCpt: 552/139 and 413, n = 9. **p<0.05, when compared to the corresponding control value, Student's t-test.

Next we determined whether Epac activation accelerates the awakening of those synaptic boutons silenced by HU-210. To this end, the cells were exposed to HU-210 for 10 min, stimulated with KCl (pulse 1 in Fig. 5A) and silent and active synaptic boutons were estimated. Then, cells were treated with the Epac activator 8pCpt and silent and active synaptic boutons were again quantified (pulse 2 in Fig. 5A). Epac treatment enhanced the average exocytotic response of the whole population of nerve terminals treated with HU-210 (Fig. 5B), largely reflecting the behaviour of the population of active synaptic boutons (Fig. 5C). However, when HU-210-induced silent synaptic boutons were considered, 8pCpt awakened 90.9±4.5% of the silent nerve terminals in contrast to the 25.9±6.4% undergoing spontaneous awakening in control (Fig 5D and E). Fig. 5F shows fluorescence images of individual nerve terminals silenced by HU-210 that, either remained silent after a second stimulation in control (upper panels), or that were awakened by exposure to the Epac activator 8pCpt (lower panels).

One important question regarding presynaptic silencing induced by HU-210 is whether this is a reversible phenomenon. We currently tested for silent synapses by stimulating granule cells with KCl (50 mM, 10 sec) immediately after incubation with HU-210 (t0). Now, we increased the time between the end of exposure to HU-210 and the test pulse. We found that increasing this time reduced the number of silent synapses. Thus, 20 min after the end of the HU-210 exposure (t20) silent synapses were reduced to 14±0.8% of that found with the standard protocol (t0) (Figure S1A). Then, presynaptic silencing induced by HU-210 is a transient event. We also explored whether longer exposure times to HU-210 increased the number of silent synapses. A 20 min incubation with 5 µM HU-210 increased the number of silent synapses by 52±0.9%, p<0.01, compared to silent synapses observed after 10 min of exposure HU-210 (Figure S1B).

We tested whether prolonged activation of cannabinoid receptors with WIN 55 212 agonist also induce presynaptic silencing. WIN 55 212 (5 µM, 10 min) strongly reduced the averaged response of nerve terminals compared to control (Figure S2A), because dramatically increased the number of silent synapses (0.49±0.49% in control and 31.7±3.93% in the presence of WIN55 212, p<0.01, Figure S2B, C).

We also tested whether CB1R activation with endocannabinoid 2-arachidonylglycerol, 2-AG, also mimics the presynaptic silencing induced by the agonist HU-210. 2-AG (60 µM, 15 min), increased silent synapses from (2.1±1.3%, n = 5) in control cells to (9.2±2.6, n = 10, p>0.5) (Figure S3A,B), although this trend do not reach statistical significance, probably due to 2-AG degradation. However, in the presence of the monoacylglycerol lipase inhibitor JZL 184 (1 µM, 30 min) 2-AG significantly enhanced the number of silent synaptic boutons (35.3±11.7, n = 8, p<0.05). Then, the endocannabinoid 2-AG also induce the presynaptic silencing observed with HU-210. JZL 184 added alone also show a statistically non significant trend to increase the number of silent synapses (11.8±4.3, n = 6, p>0.05), indicating the existence of a tonic production of endocannabinoids in the cerebellar granule cell culture in basal conditions (Figure S3A,B).

Presynaptic silencing has been associated with changes in the active zone proteins RIM (Rab3 interacting molecule) and Munc13-1 [21], [44]. Munc13 is essential for SV priming [45] and its activity is regulated by Ca^2+^, diacylglycerol, calmodulin [46] and by RIM proteins. Homodimerization of Munc13 inhibits its priming function and RIMs activate priming by disrupting Munc13 homodimerization [47]. Another important function of RIM is to bind to N- and P/Q-type Ca^2+^ channels. Through this interaction RIM proteins tether Ca^2+^ channels to the AZ [40], thereby tightly coupling Ca^2+^ influx to the triggering of vesicle fusion. Thus, the loss of RIM proteins from synapses leads to a selective loss of Ca^2+^ channels from presynaptic specializations [40]. Moreover, the decrease in the number SVs close to the plasma membrane induced by HU-210 resembles that observed in mice lacking RIM proteins [40]. Based on these observations, we investigated the correlation between the RIM1α and Munc13-1 protein levels and neurotransmitter release capacity at individual nerve terminals in FM1-43 experiments.

FM1-43 is not a good tracer of all modes vesicular fusion as vesicular retention of the fluorescent membrane marker is observed when release occurs via “kiss-and-run” [25], [26]. Thus, a failure in dye uptake due to synaptic vesicles undergoing “kiss-and-run” mode of fusion can be confounded with silent synapses. Despite these limitations, FM1-43 experiments are useful to detect an ample range of release capacities in terms of extent of FM1-43 release at individual nerve terminals [31]. In addition, FM1-43 experiments yield a higher number of exocytotic responses in a single experiment, making this technique appropriated to perform high-level statistics with thousand of nerve terminals. With these limitations in mind, we study in FM1-43 experiments whether a correlation exists between the RIM1α and Munc13-1 protein levels and neurotransmitter release capacity at individual nerve terminals.

Cells loaded with FM1-43 (as shown in Material and Methods) were treated with HU-210 (5 µM, 10 min) and then FM1-43 release induced by a short (10 sec) and more prolonged (2 min) KCl depolarizations were measured at individual nerve terminals. Subsequently, *post-hoc* immunocytochemical experiments were performed to identify synaptic proteins using antibodies against RIM1α, Munc13-1 and CB1R.

**Figure 6:**
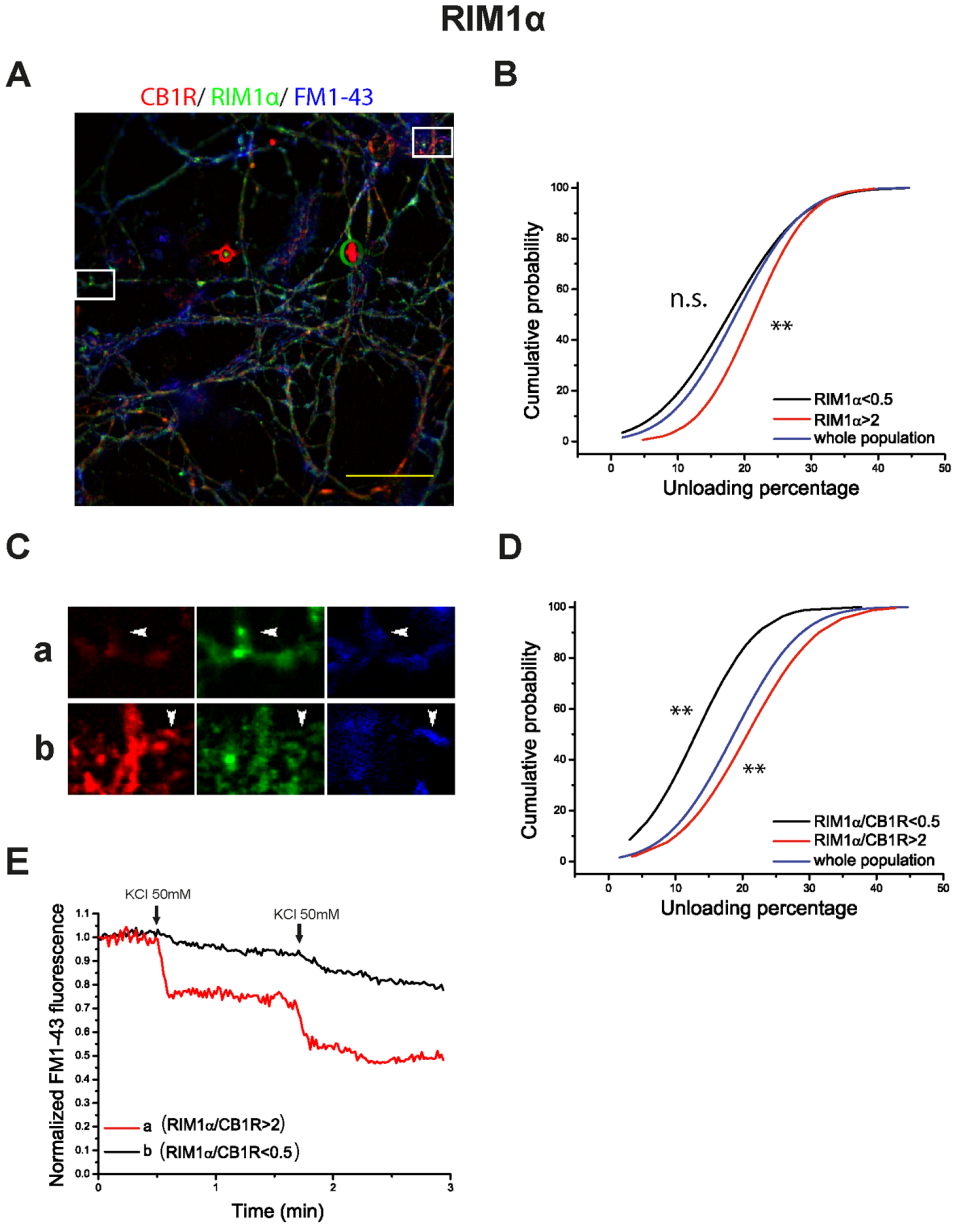
RIM1α/CB1R ratio levels determine synaptic efficiency in HU-210 treated cells. A) Post hoc immunocytochemical images of FM1-43 loaded synaptic boutons in blue (pseudocolor), anti-CB1R in red and anti-RIM1α in green. Selected areas in A are shown at higher magnification in C. Normalized FM1-43 unloading of synaptic boutons according to: RIM1α (B) and RIM1α/CB1R ratio (D) (whole population in blue; subpopulation with protein or ratio value >2 in red; subpopulation with protein or ratio value <0.5 in black). E) examples of FM1-43 unloading kinetics of the individual synaptic boutons shown in C. Traces in B are means of n = 541 synaptic boutons from 4 covers in whole population of synaptic boutons; n = 91 in RIM1α >2; n = 219 in RIM1α<0.5. Traces in D are means of n = 541 synaptic boutons from 4 covers in whole population of synaptic boutons; n = 48 in RIM1α/CB1R >2; n = 55 in RIM1α/CB1R<0.5. Scale bar 25 µm. ^NS^p>0.05, **p<0.01 compared to whole population (Kolmogorov-Smirnov test).

**Figure 7:**
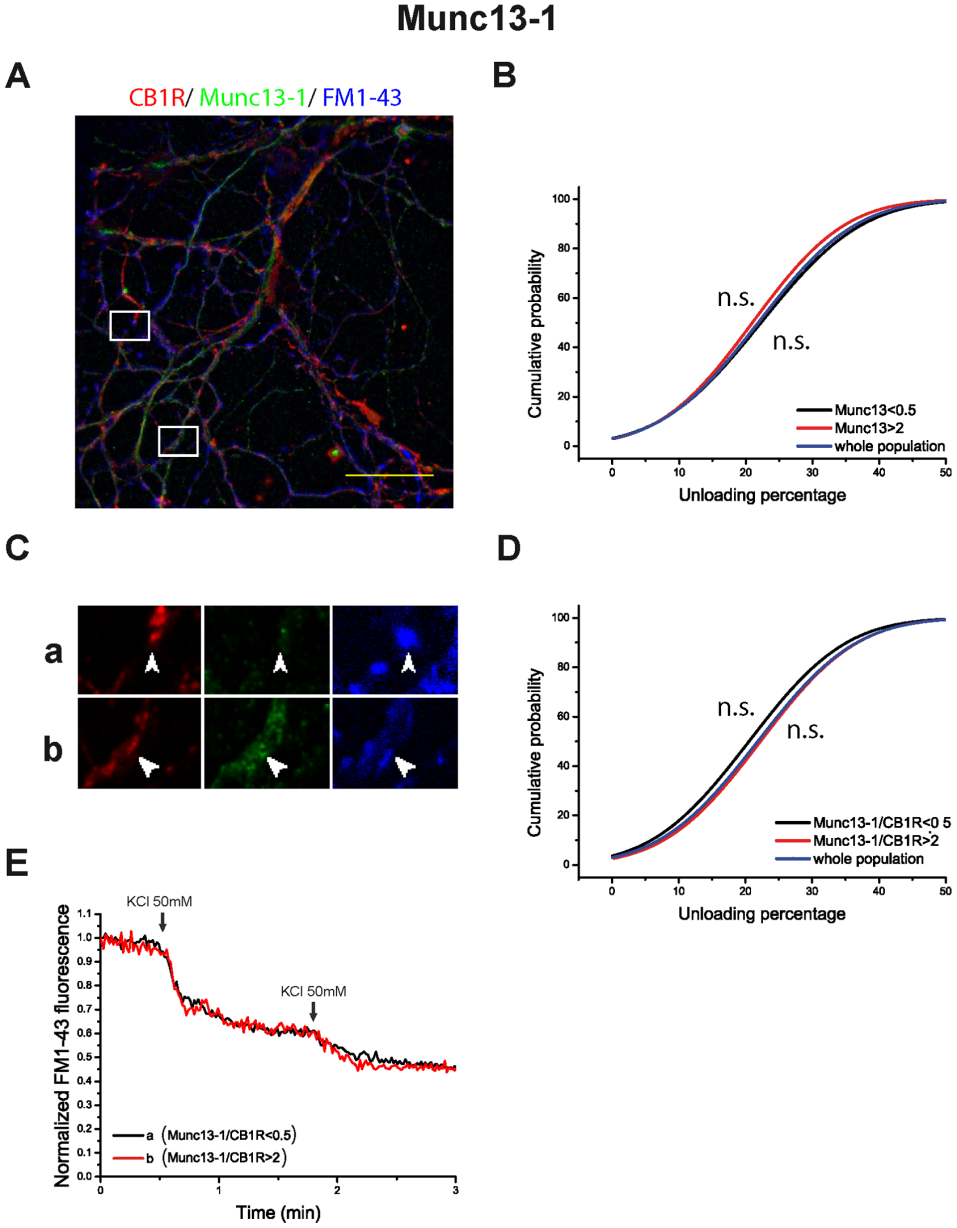
Munc13-1/CB1R ratio does not determine synaptic efficiency in HU-210 treated cells. A) Post hoc immunocytochemical images of FM1-43 loaded synaptic boutons in blue (pseudocolor), anti-CB1R in red and anti-Munc13-1 in green. Selected areas in A are shown at higher magnification in C. Normalized FM1-43 unloading of synaptic boutons according to: Munc13-1 (B) and Munc13-1/CB1 ratios (D) (whole population in blue; subpopulation with protein or ratio value >2 in red; subpopulation with protein or ratio value <0.5 in black). E) examples of FM1-43 unloading kinetics of the individual synaptic boutons shown in C. Traces in B are means of n = 3842 synaptic boutons from 4 covers in whole population of synaptic boutons. n = 594 in Munc13-1>2; n = 1951 in Munc13-1<0.5. Traces in D are means of n = 3842 synaptic boutons from 4 covers in whole population of synaptic boutons; n = 1306 in Munc13-1/CB1R >2; n = 837 in Munc13-1/CB1R<0.5. Scale bar 25 µm. ^NS^p>0.05, compared to whole population (Kolmogorov-Smirnov test).

We found that nerve terminals exhibiting a high RIM1α level display a large extent of dye release upon depolarization (Fig. 6A,B, and synaptic bouton (a) in Fig. 6C and E, as an individual example), while nerve terminals showing a low FM1-43 release also displayed low RIM1α levels (Fig. 6A,B, and synaptic bouton (b) in Fig. 7C and E, as an individual example). This correlation was even stronger when the RIM1α/CB1R ratio was examined (p<0.01, compared to whole population, Kolmogorov-Smirnov test, Fig. 6D). This data are consistent with recent reports showing that RIM protein levels are a useful marker of the release capacity of individual nerve terminals [44], [48]. By contrast, no such correlation was observed between release and Munc13-1 protein levels or the Munc13-1/CB1R ratio (Fig. 7). Thus, nerve terminals with different content in Munc13-1 protein do not exhibit a different release capacity (p>0.05, Kolmogorov-Smirnov test, Fig. 7B,D). As such, Fig. 7C and E shows individual examples of nerve terminals with different Munc13-1 protein content that exhibit in both cases a large extent of dye release. Then RIM, but not Munc13-1 protein, is a good marker of the release capacity of nerve terminals.

## Discussion

In the present study, we have analyzed the presynaptic silencing induced by the activation of cannabinoid receptors and we have drawn four principal conclusions. Accordingly, presynaptic silencing: (i) requires the prolonged (10 min) activation of CB1 cannabinoid receptors; (ii) is fully prevented by increasing cAMP levels and activation of Epac proteins; (iii) is associated with the retraction of synaptic vesicles from the active zone of the presynaptic membrane; and (iv) is associated with a low RIM1α/CB1 immunoreactivity ratio.

Presynaptically silent synapses are nerve terminals that do not release transmitter in response to a strong depolarization [18]. To distinguish true presynaptic silent synaptic boutons from those having a low release probability, we have used a strong depolarization protocol (50 mM KCl, 10 sec) known to result in a large and sustained Ca^2+^ influx in cerebellar granule cells [17], that should induce release, even at synapses with a low release probability. Indeed, with this protocol a very low content (around 1%, Fig. 2) of presynaptically silent synaptic boutons was found in cultured cerebellar granule cells at 7 DIV under control conditions. To detect cannabinoid-induced silent synapses we have used a double pulse protocol, with the cannabinoid HU-210 being applied for 10 min between the two pulses. Cannabinoid silenced synapses were those that responded to the first stimulus but failed to respond to the second one. With this approach, a 10 min treatment with CB1R agonist, silenced a 32.3±10.9% of the synaptic boutons, at 1.3 mM extracellular Ca^2+^ concentrations. Thus, CB1-induced silent synapses represent a fraction of the CB1-expressing nerve terminal in the culture of cerebellar granule cells, under these experimental conditions. Decreasing Ca^2+^ influx by reducing extracellular Ca^2+^ to 0.25 mM apparently increased the fraction of nerve terminals that underwent silencing (8.4±1.4% and 49±9%, in control and HU-210-treated cells, respectively, p<0.05, compared to control, data not shown). However, these data may not indicate an increase in the number of true silent nerve terminals, but just a failure of exocytosis of those nerve terminals with low release probability under conditions of reduced Ca^2+^ influx.

One important question refers to the signalling mechanism initiated by CB1 receptors that makes nerve terminals fail in releasing neurotransmitter. CB1R responses include the inhibition of Ca^2+^ channels [3] and inhibition of adenylyl cyclase [5], [41]. It is unlikely that the inhibition of Ca^2+^ channels mediates presynaptic silencing because increasing extracellular Ca^2+^ does not prevent silencing and because an intact Ca^2+^ influx has been associated with presynaptic silencing in cerebellar granule cells [17]. Inhibition of adenylyl cyclase reduces cAMP and this may result in a long lasting inhibition of release via changes in the release machinery. We found that CB1R-induced synaptic silencing is associated with a decrease in the cAMP levels in cerebellar granule cells and we infer that this change could be relevant to presynaptic silencing because increasing cAMP with forskolin prevented CB1R-mediated presynaptic silencing. Although, CB1R decreased basal cAMP levels by 50%, it should be noted that the cAMP measurements correspond to the whole cell compartment and that a more dramatic reduction may occur at the presynaptic level, and particularly at CB1R-expressing synaptic boutons. In addition, we found a minor role of PKA but a major relevance of Epac proteins as cAMP targets involved in preventing presynaptic silencing in cerebellar granule cells. In this context, in some presynaptic forms of long term plasticity dependent on cAMP and the active zone protein RIM1α [6], [49], the role of PKA remains controversial as presynaptic potentiation is unchanged in mice expressing a mutant form of RIM1 lacking the critical PKA phosphorylation site [50].

Certainly, PKA is not the only target of cAMP, and Epac proteins have emerged as multi-purpose cAMP receptors that specifically may have an important role in neurotransmitter release, although the Epac presynaptic targets remain largely unknown. Epac proteins are guanine nucleotide exchange factors for small GTPases that act as intracellular receptors of cAMP [43]. There are two genes and Epac1 and Epac2 proteins are widely expressed throughout the brain [51]. Several studies have shown that Epac activation enhances neurotransmitter release at excitatory central synapses [52], [53]. Furthermore, spontaneous and evoked synaptic transmission in CA1 pyramidal neurons from the hippocampus, as well as Long Term Potentiation is dramatically reduced in Epac null mutants [54]. Other reports in non-neuronal secretory systems have shown that Epac proteins promote GDP/GTP exchange on Rab3A [55] and Rap-dependent activation of PLCε and phosphatidylinositol 4-5 bisphosphate (PIP_2_) hydrolysis, thereby generating inositol 1-4-5 trisphosphate (IP_3_) and diacylglycerol (DAG) [56]. It is not known whether the Epac actions, found in other secretory systems, also occurs at central nerve terminals, but certainly they would provide pathways for functional interaction of Epac with the release machinery proteins as DAG is an activator of the active zone protein Munc13-1 essential for the priming of synaptic vesicles [45] whereas small GTPase Rab3A interacts with the active zone protein RIM (Rab3 interacting molecule) [57].

We found that synaptic boutons with low FM1-43 release after CB1R activation express low RIM1α levels. RIM proteins are active zone proteins that interact with all other known active zone proteins and with synaptic vesicles through their multiple domains [58]. RIM proteins play essential role in exocytosis through activation of vesicle priming by reversing autoinhibitory homodimerization of Munc13 [47]. Moreover, via direct and indirect interactions, RIM proteins recruit Ca^2+^ channels to the AZ [40]. Thus, presynaptic protein RIM1/2 content positively correlated with the active zone area, voltage-gated calcium channels number and release probability at hippocampal glutamatergic terminals [48]. It is possible that synaptic boutons with less RIM proteins are more susceptible to the silencing process. Interestingly, cannabinoid-induced synaptic silencing involves the retraction of SVs from the presynaptic plasma membrane, an effect also observed in conditional knockout mice lacking all RIM isoforms [40]. SV retraction should uncouple Ca^2+^ entry from exocytosis, giving rise to the presynaptic failure observed in silent synapses. Nevertheless, nerve terminals that exhibit a strong response in VGLUT1-pHluorin experiments have shown CB1R-induced silencing, indicating that nerve terminals exhibiting a large recycling pool of synaptic vesicles, are also susceptible to undergo CB1R-induced silencing. Other possible explanation is that CB1R-mediated presynaptic silencing results from RIM degradation at the proteasome [59]. It has been recently shown that the proteasome inhibitor MG132 prevents presynaptic silencing induced by chronic (4 h) depolarization of hippocampal cell cultures [21]. However, it remains to be determined whether proteasome degradation is sufficiently fast to account for the rapid silencing induced by cannabinoid agonists in cerebellar granule cells.

In contrast to RIM1α, we found that the release capacity of synaptic boutons does not correlate with Munc13-1 content. The active zone protein Munc13-1 is a phorbol ester receptor essential for the synaptic vesicle priming and for short-term potentiation of transmitter release [60]–[62]. Munc13-1 is distributed in two biochemically distinguishable soluble and insoluble pools [60], [63], [64]. Diacylglycerol and phorbol esters increase the association of Munc13-1 with the plasma membrane [65], [66]. In addition, it has been recently shown that RIM proteins activate vesicle priming by disrupting autoinhibitory homodimerization of Munc13 [47]. It is then possible, that rather to the whole protein level, release capacity is related to content of Munc13-1 heterodimer form in the proximity of the membrane compartment.

Cannabinoid-induced LTD is a widely expressed phenomenon in the brain that can be observed at excitatory synapses in different brain areas including neocortex [67], hippocampus [68], striatum [69], nucleus accumbens [70] and cerebellum [8]. Conditions required to induce cannabinoid-induced LTD are similar to that to induce presynaptic silencing at cultured cerebellar granule cells. Thus, data from hippocampus and striatum show that endocannabinoid-induced LTD requires more than 5 minutes of CB1R activation after a brief induction stimulus [71]. However, CB1R-induced presynaptic silencing in cultured granule cells is transient as most silent boutons awake after 10-20 minutes. Accordingly, it is possible that the contribution of CB1R-induced presynaptic silencing to synaptic plasticity is also transient. In this context it is important to note that cannabinoid-induced LTD at many synapses is expressed as a persistent presynaptic reduction in release. In contrast, LTD of the parallel fiber to Purkinje cell synapses is induced presynaptically, but expressed postsynaptically [9], [72], [73]. In order to determine a possible contribution of cannabinoid-induced synaptic silencing to cannabinoid-induced forms of LTD it will be necessary to apply image techniques that measure the synaptic vesicle cycle to estimate the response of individual synaptic boutons prior and after the induction of synaptic plasticity.

## Supporting Information

Figure S1 **HU-210-induced silencing is a transient phenomenon that could be magnified with longer incubation periods.** A) Percentage of silent boutons after different recovery times. Single-pulse experiments were performed after 10 min of agonist incubation and following 0, 4, 10 and 20 minutes of recovery, respectively. Then, the percentage of silent synapses was estimated (t0: 1.00±0.14, t4: 0.76±0.12, t10: 0.66±0.16, t20: 0.14±0.08, Control: 0.04±0.02). B) Comparative relation between silent synapses' percentages in control conditions and following 10 or 20 min HU-210 exposure, respectively (Control: 0.04±0.03, HU-210 10 min: 1.00±0.15, HU-210 20 min: 1.52±0.09); * p<0.05, ** p<0.01, *** p<0.001. Number of synaptic boutons analyzed (total/ silent and active synaptic boutons, n, coverslip number). A) t0: 259/51 and 208, n = 8; t4: 358/50 and 308; t10: 386/41 and 345, n = 8; t20: 200/9 and 191, n = 7; Control: 197/2 and 195, n = 7. B) Control: 197/2 and 195; t10′ 284/42 and 242, n = 8; t20′ 360/75 and 285.
(EPS)

Figure S2 **Presynaptic silencing induced by the cannabinoid receptor agonist WIN 55 212.** Control cells in HBM and WIN 55 212 (5 µM, 10 min) treated cells, were stimulated with 50 mM KCl (10 sec) followed by the addition of NH_4_Cl (50 mM). Average response of the whole population in control and WIN55 212 treated cells are shown in (A). Percentage of silenced synaptic boutons in control and WIN55 212 (B). C) representative fluorescence images of synaptic boutons responding to 50 mM KCl (10 sec) followed by NH_4_Cl (50 mM) perfusion in control (HBM) and after WIN 55 212 (5 µM, 10 min). Number of synaptic boutons analyzed (total/ silent and active synaptic boutons, n, coverslip number). Control: 229/2 and 227, n = 5; WIN55 212: 364/132 and 232, n = 7. **p<0.05, when compared to the corresponding control value, Student's t-test.
(TIF)

Figure S3 **Presynaptic silencing is also induced by endocannabinoid treatment.** A) Cells were incubated with HBM (control), the endocannabinoid 2AG (60 µM, 15 min), the inhibitor of the monoacylglycerol lipase JZL 184 (1 µM, 30 min) and then stimulated with 50 mM KCl (10 sec). B) The percentage of silent boutons in the different conditions was determined: control (2.06±1.27, n = 5), 2AG (9.23±2.56, n = 10), JZL184 (11.84±4.31, n = 6), JZL184+2AG (35.33±11.73, n = 8); *p<0.05. Number of synaptic boutons analyzed (total/ silent and active synaptic boutons, n, coverslip number). Control: 165/4 and 161, n = 5; 2AG: 407/38 and 369, n = 10; JZL184: 134/16 and 118, n = 6; 2AG+JZL184: 342/121 and 22, n = 8.
(EPS)
